# A health promoting-lifestyle prediction model for dementia prevention among chinese adults: based on the health belief model

**DOI:** 10.1186/s12889-022-14828-9

**Published:** 2022-12-28

**Authors:** Hua Li, Jinying Zhang, Li Wang, Tiantian Yang, Yanni Yang

**Affiliations:** 1 School of Nursing, Army Medical University, Chongqing, China; 2 Department of Otolaryngology Head and Neck Surgery, The 940th Hospital of Joint Logistics Support force of Chinese People’s Liberation Army, Lanzhou, China

**Keywords:** Health promoting-lifestyle, Dementia prevention, Health belief model, Chinese adults

## Abstract

**Background:**

People’s health belief is an important factor affecting health behavior. However, there has been little use of the health belief model (HBM) in determining the pathway effect of patients’ beliefs on health behavior in dementia prevention in China. The aim of our study was to evaluate the impact of dementia prevention beliefs on health promoting lifestyle among Chinese adults.

**Methods:**

A cross-sectional study was conducted on line by convenience sampling from January to March 2020. A survey about dementia prevention knowledge, health belief of dementia prevention and health-promoting lifestyle was completed by 1201 adults in China. Data was analyzed using a structural equation model with the analysis of moment program.

**Results:**

The participants were aged 40.50 ± 12.72 years. About 70.3% of participants were female. The purposed model fit the data from the study well. Perceived barriers (total effect-0.322, *P* < 0.01) and perceived susceptibility (total effect -0.242, *P* < 0.01) had negative effects on lifestyle. Self-efficacy had promoting effects on lifestyle (total effect 0.207, *P* < 0.01). Perceived severity had positive effects both on perceived benefits (total effect 0.137, *P* < 0.01) and perceived barriers (total effect 0.202, *P* < 0.01), which had a contradictory effect in the formation of health belief. Perceived benefits, cues to action and self-efficacy played a partial mediating role between knowledge and health behavior. The belief of changing lifestyle to reduce the risk of dementia could explain 24.5% of health behavior (*P* < 0.05).

**Conclusions:**

The findings indicate that in dementia prevention, dementia prevention health belief has important influences on health behavior. Community medical staff can develop targeted dementia prevention interventions based on the health belief model in the future.

## Introduction

Dementia is a kind of neurodegenerative syndrome, which seriously affects the daily life of individuals and has become an important cause of disability in the elderly [1]. Dementia is a rapidly increasing public health problem affecting about 50 million people around the world. There are nearly 10 million new cases every year and this figure is set to triple by 2050. China has become the country with the largest number of dementia patients. It is estimated that by 2030, the number of dementia patients over 60 years old in China will reach more than 23 million [2]. Since there is still a controversy over drugs to prevent and reverse dementia, prevention of controllable factors for dementia is the most cost-effective measure. The importance of dementia prevention has been further highlighted with the release of the WHO’s 2019 Guidelines for The Risk Reduction of Cognitive Decline and Dementia [3].

Due to severe cognitive impairment in advanced dementia, individuals with dementia will lose their basic living ability and lead to inability to take care of themselves. The stigma of dementia in society has not been completely eliminated, causing the different cognition of people between dementia and other chronic diseases. Akenine et al. [4] compared the differences of people’s attitudes towards cardiovascular disease and dementia, it has been found that people think that cardiovascular disease has an expectable prognosis, while dementia is almost impossible to cure, and people often associate fear, shame and despair with dementia.

Dementia is not an inevitable consequence of ageing, while age is the strongest known risk factor for cognitive decline. Several recent studies have shown a relationship between the development of cognitive impairment and dementia with lifestyle-related risk factors, such as physical inactivity, tobacco use, unhealthy diets and excessive drinking [5–7]. Many countries have carried out exploratory researches on population-based dementia prevention and intervention. The results show that following a healthy lifestyle can significantly improve individual’s cognitive function and help to delay or reduce the risk of dementia [8].

The health belief model (HBM) has been continuously developed and improved for decades as a widely used health behavior theory to examine the barriers and formation of a person’s participation in programs which focus on prevention of disease or promotion of a healthy lifestyle. It has been used and confirmed in the self-management of chronic diseases [9], cancer screening behavior [10, 11], prevention of communicable disease [12] and other health-related behaviors. The HBM was effectively used to explain and predict the formation and maintenance of behavior from the personal perspective. So, we speculated that the health belief model also can be used to explain the prevention behavior of dementia. The health belief model can be used to explain and predict health behavior, but the effects of dimensions on behavior are different in different diseases and behavior types [10, 11]. Health behavior can be divided into many types, such as preventive behavior, disease screening and patient role behavior. For different types of behavior, the effect of each dimension of health belief is varying. Based on previous studies, in preventive behavior, perceived benefits, perceived barriers and self-efficacy play a significant role, and in disease screening behavior, perceived susceptibility has the strongest effect on health behavior [13]. The correlation between perceived severity and behavior is weak, or even there is no significant correlation in other behaviors besides patients’ role behaviors [14]. So, for dementia prevention, it’s necessary to construct the interaction between the various dimensions of health belief in dementia prevention, and to clarify the effects of each dimension on health behavior, so as to determine the mechanism of health belief in dementia prevention.

Dementia related risk factors are gradually confirmed, but how to transform the existing evidence into people’s daily behavior still needs more exploration. Based on the health belief model, the purposes of this study are to (1) describe the levels of health beliefs and health behaviors associated with the dementia prevention in Chinese adults and (2) validate the relationship between the dimensions of health beliefs and their predictive effect on health promotion lifestyles by using structural equation model.

## Methods

A cross-sectional study was conducted on line from January to March 2020. Eligible adults were recruited from China using convenience sampling. The inclusion criteria were: (1) able to read, comprehend and complete questionnaires; (2) age ≥ 18 years; (3) informed consent and voluntary participation in this study. The exclusion criterion was: people who completed questionnaires improperly (e.g., all items selected the same option or the filling time was less than 300 s).

According to the sampling calculation formula: N = [u_α/2_ × σ/δ]^2^. (α = 0.05, u_α/2_ = 1.96).The sample size was determined by the scale with the highest number of items, among which the Health Promoting Lifestyle Profile-II, Revise(HPLP-II R) scale has the highest number of items, σ taking a value of 15.66 for the previous research [15], the HPLP-II R has a highest score of 160, the allowable error δ could take a value of 1, and the calculated sample size was about [1.96 × 15.66/1]^2^ = 942. Considering that there may be 20% of invalid questionnaires, a final sample size of 1130 cases was calculated.

The online questionnaire and its link were created on a common platform for online survey in China. From January to March 2020, data collector publicized the link of questionnaire on WeChat (an online socializing platform), and explained the purpose, content, as well as methods and notes to the respondents. The questionnaire was carried out anonymously in accordance with the voluntary principle. After completing and submitting the questionnaire, the data were automatically uploaded to the platform. All data could be exported through the platform. Participants could only submit once through each IP address to avoid repeated submission, and all questions must be answered before submission. A total of 1202 questionnaires were collected in this study. One questionnaire with obvious errors (all items were selected with the same option) was removed, and 1201 valid questionnaires were finally recovered, with an effective recovery rate of 99.9%, and the sample size could meet the needs of our study. This study was approved by the Ethics Committee of the Army Medical University, and complied with ethical guidelines and regulations according to the principles of the Declaration of Helsinki.

## Measures

### Demographic characteristic questionnaire

The questionnaire covered the demographic characteristics of participants, including sex, age, province, residential address, educational level, work status, marital status, as well as contact history and family history of dementia.

### Dementia prevention knowledge questionnaire

The questionnaire was self-designed based on Risk Reduction of Cognitive Decline and Dementia by WHO to assess the knowledge of dementia prevention. A total of 11 questions were included in this questionnaire. These items concerned self-reported knowledge of modifiable dementia risk and protective factors (hypertension, smoking, physical activity, nutrition, mental activity, depression and diabetes mellitus).

### Motivation to Change Lifestyle and Health Behaviors for Dementia Risk Reduction Scale (MCLHB-DRR)

The MCLHB-DRR was developed by Kim et al. [16]. to measure people’s health belief of dementia prevention. This scale was consisted of 27 items in 7 domains, including perceived susceptibility (4 items, for example, chances and possibility of developing dementia), perceived severity (5 items, for example, the thought of dementia scares me, when I think about dementia my heart beats faster, when I think about dementia I feel nauseous), perceived benefits (4 items, for example, changing my lifestyle and health habits can help me reduce my chance of developing dementia, I have a lot to gain by changing my lifestyle and health behavior, adapting to a healthier lifestyle and behavior would prevent dementia for me), perceived barriers (4 items, for example, I am too busy to change my lifestyle and health habits, my financial situation does not allow me to change my lifestyle and behavior, changing lifestyle and behavior interferes with my schedule), cues to action (4 items, for example, having risk factor(s) for dementia makes me think I have to change my lifestyle and behavior, learning more about dementia from the media makes me think I have to change my lifestyle and behavior, knowing family member(s) with dementia makes me think I have to change my lifestyle and behavior), general health motivation (4 items, for example, nothing is as important to me as good health, I often think about my health, I think I have to pay attention to my own health) and self-efficacy (2 items, for example, I am certain that I can change my lifestyle and behavior so I can reduce the risk of developing dementia.). A 5-point Likert scale from 1 (strongly disagree) to 5 (strongly agree) was used in each item. While the items of Perceived Barriers were scored in reverse. The sum of the scores of all items in the scale ranged 27–135, with higher scores indicating higher level of motivation to reduce the risk of dementia. It was reported that the Cronbach alpha values of 7 domains ranged from 0.608 to 0.864 [16].

In a previous study, the scale has been translated into Chinese version through forward and backward translation according to the guidance of Bristlin (Brislin, 1970), and the validity and reliability of it has been tested [17]. Results showed that the Chinese version of this scale has an acceptable structure validity with factor loadings of each item ranging from 0.406 to 0.944, as well as a good retest reliability as 0.868 and the Cronbach’s alpha as 0.763. In this study, the Cronbach’s alpha was 0.830.

### Health Promoting Lifestyle Profile-II, Revise (HPLP-II R)

The HPLP was developed by Walker et al. [18] to assess the health promoting lifestyle of people in 1987, and was revised as HPLP-II in 1997. Then, it was translated into several versions in other countries to satisfy the local culture. In China, it was translated and revised as HPLP-II R by Cao et al. [19] in 2014. There were 40 items within 6 domains in the HPLP-II R, including nutrition (6 items, for example, eat breakfast, fruit and vegetables every day), sports (8 items, for example, do stretching exercise, check pulse rate, vigorous exercise 3 times/week), health and responsibility (11 items, for example, read books about health, discuss health concerns, discuss health issues with professionals), interpersonal relationships (5 items, for example, praise other easily, maintain meaningful interpersonal relationships, enjoy touching), spiritual growth (5 items, for example, look forward to future, know what is important, life has purpose) and stress management (5 items, for example, pleasant bedtime thoughts, use stress control methods, relaxation). A 4-point Likert scale from 1 (never) to 4 (always) was used in each item. The sum of the scores of all items in the scale ranged 40–160, with higher scores indicating higher level of health promotion lifestyle. It was reported that the Cronbach’s alpha of the HPLP-II R and its domains were from 0.84 to 0.91 [19]. In this study, the Cronbach’s alpha of it was 0.933.

### Statistical analysis

**Fig. 1:**
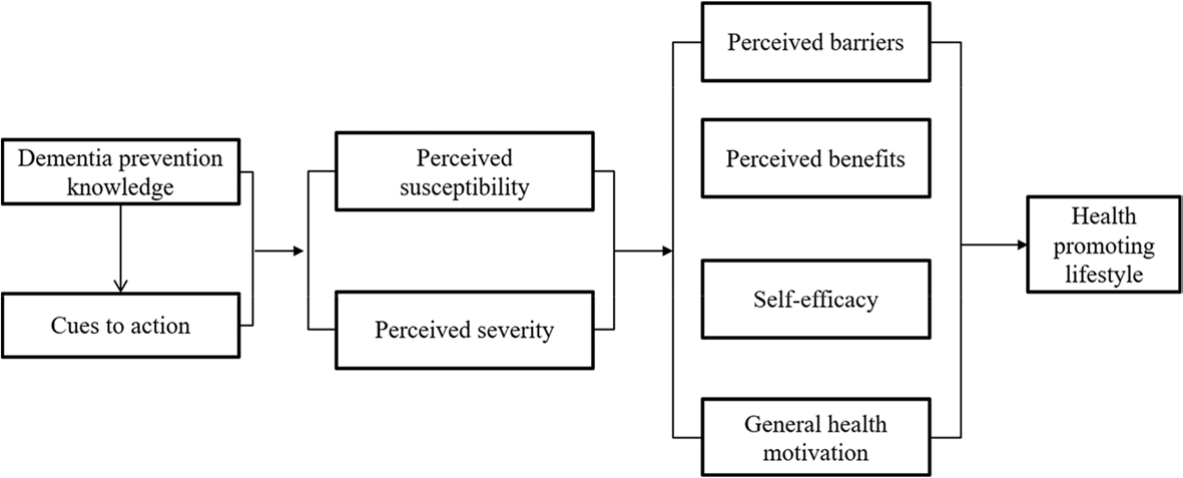
Proposed integrative behavior theory derived model for understanding factors predicting health promoting lifestyle in dementia prevention

Descriptive statistics and Pearson correlation coefficients were computed for all study variables by utilizing the SPSS24.0. Based on previous studies, a proposed model was designed (Fig. 1). The AMOS 24.0 was used to establish the structural equation model to evaluate the fitting index of the proposed model. The proposed model would be accepted only if test values satisfied the following standards: χ^2^/df < 3, GFI/AGFI/CFI > 0.9, RMSEA < 0.05. Otherwise, it would be revised based on experts’ discussions and retested through the structural equation model.

## Results

### Demographics and dementia-related characteristics

There were 1201 Chinese adults included in this study. Demographics and dementia-related characteristics of the subjects are presented in Table 1. The mean age of respondents was 40.50 ± 12.72 years. About 70.3% of participants were female, and most of participants (86.5%) were living in the city. Most of the subjects (59.1%) had bachelor degree or above. About 28.3% of the respondents had come into contact with dementia patients.

**Table 1:** General and dementia-related characteristics of the participants (*n* = 1201)

Variables	Categories	N(%)
Gender	Male	357 (29.73)
	Female	844 (70.27)
Resident	Rural	162 (13.49)
	City	1039 (86.51)
Level of education	Middle school and below	93 (7.74)
	High school	186 (15.24)
	Junior college	213 (17.74)
	University	564 (46.96)
	Master’s degree and above	145 (12.07)
Occupation	Yes	1011 (84.18)
	No	190 (15.82)
Marital status	Married	883 (73.52)
	Single	274 (22.81)
	Divorced, bereaved	44 (3.66)
Underlying chronic non-communicable diseases	No	939 (78.18)
	Yes	262 (21.82)
Family history of dementia	No	1104 (91.92)
	Yes	97 (8.08)
Contact with dementia patients	No	861 (71.69)
	Yes	340 (28.31)

### Descriptive statistics regarding the characteristics of the measured variables

The range of the observed variables’ skewness and kurtosis were 0.169—2.010 and 0.263—4.698, respectively, which signified a normal distribution (Table 2).

**Table 2:** Descriptive statistics of measured variables (*n* = 1201)

Variables	Score(M ± SD)	Skewness	Kurtosis
General health motivation	17.58 ± 3.13	-2.010	4.698
Self-efficacy	8.12 ± 1.71	-1.122	1.517
Perceived benefits	16.33 ± 3.63	-1.355	1.816
Cues to action	14.62 ± 3.76	-0.653	0.263
Perceived severity	13.74 ± 4.82	0.169	-0.393
Perceived barriers	7.63 ± 2.35	0.179	-0.774
Perceived susceptibility	8.32 ± 3.29	0.225	-0.701
Health promotion lifestyle	100.00 ± 15.81	0.328	0.449

Correlation analysis supported the relationship among the study variables in the proposed model, as reported in Table 3. All dimensions of health belief were significantly correlated with health behaviors. Most dimensions of health belief interacted with each other.

**Table 3:** Correlation of measured variables (*n* = 1201)

	1	2	3	4	5	6	7	8
1.Perceived susceptibility	1							
2.Perceived severity	0.369^**^	1						
3.Perceived benefits	-0.092^**^	0.214^**^	1					
4. Perceived barriers	0.251^**^	0.267^**^	-0.012	1				
5. Cues to action	0.082^**^	0.302^**^	0.511^**^	0.082^**^	1			
6.General health motivation	-0.096^**^	0.132^**^	0.521^**^	-0.033	0.487^**^	1		
7. Self-efficacy	-0.189^**^	0.035	0.476^**^	-0.120^**^	0.466^**^	0.664^**^	1	
8.health promotion lifestyle	-0.241^**^	-0.107^**^	0.242^**^	-0.361 ^**^	0.151^**^	0.266^**^	0.341^**^	1

^**^ *P* < 0.01

^*^ *P* < 0.05

### Test of the proposed model

**Fig. 2:**
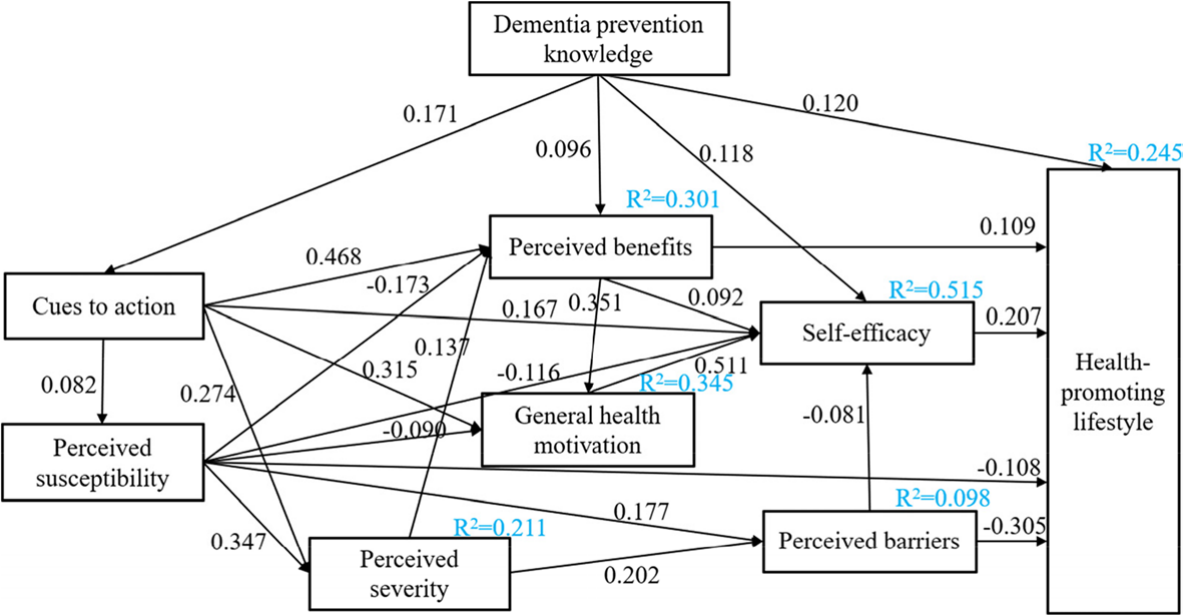
The final model. Note. Figure reports only significant paths (*n* = 1201)

The proposed model was tested based on HBM, and the final model fit was adequate (Fig. 2), as indicated by the following value: χ^2^/df = 2.974 (which was less than 3), GFI = 0.997, AGFI = 0.978, CFI = 0.994, SRMR = 0.0190, and RMSEA = 0.041. All the parameters satisfied the acceptance criteria, indicating a robust fit.

### Effect estimate

For the analysis of results regarding the model’s coefficients, Table 4 displayed the significant pathways for participants. For variables influencing health promotion lifestyle, direct effects of prevention knowledge (β = 0.120), self-efficacy (β = 0.207), perceived barriers (β = -0.305), perceived susceptibility (β = -0.108) and perceived benefit (β = 0.109) were significant, while cues to action (β = 0.114), perceived severity (β = -0.042) and general health motivation only have indirect effects on health promotion lifestyle. Self-efficacy has the greatest positive impact on lifestyle (β = 0.207), while perceived barriers (β = -0.322) and perceived susceptibility (β = -0.242) have a negative impact on lifestyle.

**Table 4:** Standardized direct, indirect and total effects of modified model (*n* = 1201)

Endogenous variables		Standardized direct effect	Standardized indirect effect	Standardized total effect
Cues to action	Dementia prevention knowledge	0.171^**^	—	0.171^**^
Perceived susceptibility	Dementia prevention knowledge	—	0.014^**^	0.014^**^
	Cues to action	0.082^**^	—	0.082^**^
Perceived severity	Dementia prevention knowledge	—	0.052^**^	0.052^**^
	Cues to action	0.274^**^	0.028^**^	0.302^**^
	Perceived susceptibility	0.347^**^	—	0.347^**^
Perceived barriers	Dementia prevention knowledge	—	0.013^**^	0.013^**^
	Cues to action	—	0.075^**^	0.075^**^
	Perceived susceptibility	0.177^**^	0.070^**^	0.247^**^
	Perceived severity	0.202^**^	—	0.202^**^
Perceived benefits	Dementia prevention knowledge	0.096^**^	0.084^**^	0.180^**^
	Cues to action	0.468^**^	0.027^**^	0.495^**^
	Perceived susceptibility	-0.173^**^	0.047^**^	-0.126^**^
	Perceived severity	0.137^**^	—	0.137^**^
General health motivation	Dementia prevention knowledge	—	0.116^**^	0.116^**^
	Cues to action	0.315^**^	0.167^**^	0.481^**^
	Perceived susceptibility	-0.090^**^	-0.044^**^	-0.134^**^
	Perceived severity	—	0.048^**^	0.048^**^
	Perceived benefits	0.351^**^	—	0.351^**^
Self-efficacy	Dementia prevention knowledge	0.118^**^	0.102^**^	0.220^**^
	Cues to action	0.167^**^	0.276^**^	0.443^**^
	Perceived susceptibility	-0.116^**^	-0.100^**^	-0.216^**^
	Perceived severity	—	0.021	0.021
	Perceived barriers	-0.081^**^	—	-0.081^**^
	Perceived benefits	0.092^**^	0.180^**^	0.271^**^
	General health motivation	0.511^**^	—	0.511^**^
Health promotion lifestyle	Dementia prevention knowledge	0.120^**^	0.060^**^	0.180^**^
	Cues to action	—	0.114^**^	0.114^**^
	Perceived susceptibility	-0.108^**^	-0.134^**^	-0.242^**^
	Perceived severity	—	-0.042^**^	-0.042^**^
	Perceived barriers	-0.305^**^	-0.017^**^	-0.322^**^
	Perceived benefits	0.109^**^	0.056^**^	0.165^**^
	General health motivation	—	0.106^**^	0.106^**^
	Self-efficacy	0.207^**^	—	0.207^**^

^**^ *P* < 0.01

^*^ *P* < 0.05

## Discussion

This study aimed to determine the impact of dementia prevention beliefs on health behaviors. Results of our research demonstrated that health belief and knowledge of dementia prevention could explain 24.5% of the total variation of health promoting lifestyle. Zeng Yongjun et al. [20] found that the dimensions of the health belief model can explain 51.3% of the total variation of chronic disease prevention behavior, indicating that the health belief model can effectively explain and predict chronic disease prevention behavior. However, in our study, the dimensions of MCLHB-DRR constructed based on the health belief model could only explain 24.5% of the health promoting lifestyle. The main reason might be that people's cognitive level of disease determines whether they have corrected and positive health beliefs, while the respondents' cognitive level of dementia prevention was low [21]. The dimensions of health belief played different roles in health promoting lifestyles. Perceived barriers and susceptibility were the main barriers to health promoting lifestyle, while self-efficacy, perceived benefits and cues to action were the promoting factors. These findings had several theoretical and implications, as discussed below.

Perceived barriers refer to people’s awareness of the difficulties in taking action. In this study, perceived barriers had the greatest direct impact on lifestyle. In line with previous study, Becker et al. [14] found that in many types of health behaviors, perceived barriers have been always found as the most negative factor for behavior. The more difficulties and obstacles people felt in behavior change, the worse it was for them to adopt healthy behavior and lifestyle. The more difficulties and barriers that people felt in behavior change, the less conducive to their compliance with health behaviors and lifestyles. Therefore, in the process of dementia prevention intervention, reducing people’s perceived barrier is the key factor to improve the prevention belief of dementia and promote the development of healthy behavior habits.

It should be noted that perceived susceptibility of dementia also had a negative effect on lifestyle, indicating Chinese adults think that the higher the risk of dementia is and the less healthy lifestyles are. On the one hand, it had a direct negative impact on lifestyle; but on the other hand, it had an impact on behavior through the mediation of perceived benefits, perceived barrier, self-efficacy and perceived severity. The higher the risk people perceived, the less conducive to the development of their healthy behavior. In prevention of disease, perceived susceptibility played an important role [14]. In the application of health belief model in a variety of diseases, it was found that perceived susceptibility played a positive role in most health-related behaviors [10, 22], and some studies showed negative effects on health behavior [23]. In this study, the negative effects of perceived susceptibility might be related to characteristics of dementia. People believes that the incidence of dementia is determined by genes, and fatalism plays a dominant role in perceived susceptibility. The study indicated that people with a family history of dementia would think that they had higher morbid risk [24]. In the analysis of influencing factors, a previous study also indicated that the family history of dementia and the experience of contact with dementia patients increased people’s uncertainty about dementia prevention [25], which was not conducive to the formation of their health behaviors. Moreover, people are often not aware of the risk factors of dementia. According to the world Alzheimer's report in 2019, only one-quarter of the respondents correctly recognized that dementia is preventable [26]. Due to the lack of professional health guidance, most people still hold the opinion that dementia is non preventable [27]. Sufficient knowledge reserve is the basis of establishing a correct belief system. If people think that such diseases are uncontrollable, they will not consider that health behaviors are beneficial to dementia prevention, nor will they take further actions to prevent dementia. In the survey of the intention to early screen of dementia in Japanese elderly people, it was found that people’s awareness of dementia susceptibility promoted the formation of their screening intention [28]. It can be seen that even in the same disease, perceived susceptibility plays different roles in different behavior types. Therefore, in the future health education on dementia prevention, we should strengthen the controllability and preventability of dementia. If people think this kind of disease can be prevented and controlled, susceptibility may have a positive effect on healthy behavior. The results of this study also suggest that, due to the difference of susceptibility to health behaviors, the separate analysis by each dimension can be more meaningful than analysis by total score.

An important finding of this study was that self-efficacy was the most important direct factor in promoting healthy behavior. Self-efficacy represents the confidence of people to give up or implement certain health behaviors, and is an important part of several health behavior theories. In this study, self-efficacy was the confidence of following healthy lifestyle to reduce dementia risk, which was an important mediator among cues to action, perceived susceptibility, perceived benefits and lifestyles. It was directly influenced by other dimensions of health beliefs and had a direct effect on lifestyle. Sheeran et al. [29] reviewed previous studies and pointed that in a variety of health behavior theories, the effect of self-efficacy on behavior change was moderate, and it was a factor that had greater impact compared with social norms, attitudes, etc. Intervention on self-efficacy also achieved remarkable results in the management of blood glucose and the improvement of quality of life in diabetic patients [30]. In research on drug addiction treatment based on the health belief model, it was found that self-efficacy not only played an important intermediary role in perceived benefit, perceived barriers and drug addiction abstinence, but also was the only factor that has a long-term effect, which was not only conducive to behavior change, but also could promote the maintenance of healthy behavior [31]. Therefore, the important role of self-efficacy in health behavior promoting deserves our further attention in research and intervention.

Another interesting finding of this study was that general health motivation was an important promoting factor of self-efficacy, and it had an indirect positive impact on lifestyle through the completed mediating role of self-efficacy. The results showed that the more people paid attention to their general health, the more confident they were to reduce the risk of dementia by changing their lifestyles. The promotion effect of general health motivation on health behavior was consistent with previous research, but the effect size was different. Peng Huijiao et al. [32] found that general health motivation is the belief factor that plays the most important role in the health behavior of stroke patients. In this study, it had little effect on health behavior through the mediating role of self-efficacy, which showed that the general health motivation has different effects on different diseases. Meanwhile, improving individual’s attention to the overall health condition and not only focusing on specific diseases, promoting healthy behavior to accelerate overall health is probably one of the effective ways to reduce the incidence rate of dementia.

Interestingly, perceived severity refers to the individual’s perception of the seriousness of a kind of disease, including people’s judgment of the clinical consequences caused by the disease (such as pain, disability, death, etc.) and the social consequences caused by the disease (such as work worries, unemployment, family and social relations affected, etc.), which played a contradictory effect in the formation process of dementia prevention beliefs. On the one hand, perceived severity was an important direct factor to enhance perceived barriers, and its direct effect was stronger than perceived susceptibility. On the other hand, in the impact of perceived benefits, the total effect of perceived severity and susceptibility was just the opposite. The role of perceived susceptibility was to weaken perceived benefits, while severe perception was the promotion of perceived benefits. Thus, although the total effect on behavior was modest and ultimately negative, perceived severity could both negatively affect health behaviors by promoting perceived barrier and positively affect health behaviors by promoting benefit perceptions, leading to paradoxical effects on behavior that deserve our attention. In addition, perceived susceptibility and cues to action could affect health behavior through the indirect effect of perceived severity, also had a contradictory effect on healthy behavior. Werner’s study showed that excessive worry about dementia harms to individuals’ physical and mental health [33], while Kim [34] found that worry about the risk of developing dementia can promote the development of healthy behavioral habits. Perceived severity and susceptibility to dementia represent the cognitive level about prevalence and outcomes of dementia, and also reflect the level of concern about it. At present, individual’s perceived threats of dementia did not promote healthy behavior, but played a certain role in promoting health belief. Future research should clarify the specific connotation of severity perceptions, reinforce their positive acting portion and weaken the negative portion at the time of intervention, so that perceived severity can play the profitable role in promoting health behaviors. The negative effect of perceived severity on health behaviors was weak and consistent with previous findings by Becker [14]. After reviewed multiple studies based on the health belief model, they found that perceived severity had minimal effect on disease prevention behaviors, but the significant findings of this study suggest that the complex contradictory effects that perceived severity exerts on other dimensions of health beliefs cannot be ignored [14]. When formulating an intervention program aimed at improving dementia prevention beliefs, we need to consider the dual effects of perceived severity, and make rational intervention to achieve the best effect of promoting healthy behavior.

Cues to action, perceived benefits and self-efficacy played partial mediating roles between dementia prevention knowledge and health behaviors. It was known from the final path analysis that knowledge of dementia prevention not only had a directly impact on lifestyle, but also produced through the mediating role of cues to action, perceived benefit and self-efficacy in health beliefs. A previous study once applied the health belief model to explain stroke prevention behaviors among hypertensive patients, and the findings revealed that health beliefs among hypertensive patients play a completely mediating role in knowledge and behaviors [35]. We found that the knowledge of dementia prevention can not only directly affect health behavior, but also indirectly work through the effect of some dimensions of health belief. Our results indicated that the knowledge of dementia prevention has no direct effect on perceived barriers, perceived susceptibility and perceived severity, and the indirect effect is more complicated. Cues to action had no direct effect on healthy behavior, but could directly strengthen people's belief such as perceived susceptibility, perceived severity, perceived benefits and self-efficacy, and ultimately promoted the development of healthy behavior. A previous study also showed that cues to action play a role in intentions of weight management through perceived threat of illness [22]. The relationships between knowledge, each dimension of health beliefs and behaviors are changing with different diseases and population characteristics. Clarifying the function routes of knowledge and health beliefs is beneficial for more rational design of dementia prevention intervention programs to achieve optimal intervention outcomes.

Health behavior plays an important role in the prevention and management of chronic diseases. Behavioral interventions are mostly nurse-led, and programs established based on a rational theoretical framework promote interventions is more precise and effective. The health belief model has been one of the top three most popular and applied health behavior theories over the past decades (the other two are theories of social cognition and transtheoretical models). Health behaviors encompass a variety of behavioral categories such as preventive behaviors, disease screening behaviors, and patient’s role behaviors, The mechanism of health belief varies in different behavior types [30].The health belief model is founded based on motivation theory, cognition theory, and value expectation theory that emphasize the dominant role of subjective psychological processes on behavior [31]. Subjective psychology include multiple aspects such as cognition, emotion, and volition, and various among different populations [31]. The complexity and diversity of psychological processes resulting from different disease characteristics may have different effects on the mechanisms of health beliefs.

This study had a number of limitations. The most important was that this study adopted convenient sampling. In the case of full respect for the informed consent of the respondents, the sample size and sample distribution were limited, and the representativeness might be insufficient. Another limitation of this study was that the dementia-specific health behavior scale is not used, because WHO only published the Guideline of the Risk Reduction of Cognitive Decline and Dementia in 2019, and no dementia-specific health behavior scale has been found yet. Furthermore, this study used a cross-sectional online survey, and intervention studies may be needed to verify the effect of health belief model in prevention of dementia in the future.

## Conclusion

In conclusion, this study clarified the effect of health belief on healthy promoting behavior in dementia prevention. The results showed that perceived susceptibility, perceived barriers and self-efficacy were important targets for future dementia prevention interventions. Perceived susceptibility and severity of dementia had a negative effect on healthy lifestyle. People believed that the higher the risk of dementia, the less conducive to the formation of healthy behavior. Dementia prevention knowledge can not only directly affect health behavior, but also affect health-promoting lifestyle through the intermediary role of cues to action, perceived benefit and self-efficacy. Therefore, it is necessary to make people aware that dementia is a preventable and controllable disease, and dementia-related health education is imminent for Chinese adults.

## Data Availability

The datasets used and/or analyzed during the current study available from the corresponding author on reasonable request.
